# Colloidal Self-Assembly of Inorganic Nanocrystals into Superlattice Thin-Films and Multiscale Nanostructures

**DOI:** 10.3390/nano9091243

**Published:** 2019-09-01

**Authors:** Hongseok Yun, Taejong Paik

**Affiliations:** 1 Department of Chemical and Biomolecular Engineering, Korea Advanced Institute of Science and Technology (KAIST), Daejeon 34141, Korea; 2 Department of Integrative Engineering, Chung-Ang University, Seoul 06973, Korea

**Keywords:** BNSL, superlattice, self-assembly, colloidal nanocrystal, binary nanocrystal superlattice

## Abstract

The self-assembly of colloidal inorganic nanocrystals (NCs) offers tremendous potential for the design of solution-processed multi-functional inorganic thin-films or nanostructures. To date, the self-assembly of various inorganic NCs, such as plasmonic metal, metal oxide, quantum dots, magnetics, and dielectrics, are reported to form single, binary, and even ternary superlattices with long-range orientational and positional order over a large area. In addition, the controlled coupling between NC building blocks in the highly ordered superlattices gives rise to novel collective properties, providing unique optical, magnetic, electronic, and catalytic properties. In this review, we introduce the self-assembly of inorganic NCs and the experimental process to form single and multicomponent superlattices, and we also describe the fabrication of multiscale NC superlattices with anisotropic NC building blocks, thin-film patterning, and the supracrystal formation of superlattice structures.

## 1. Introduction

In the past decades, colloidal inorganic nanocrystals (NCs) have received considerable attention in several research fields because of their interesting size-dependent properties, such as their quantum confinement effect and localized surface plasmonic effect, which are not be observed in the bulk [1,2]. With extensive research efforts, there has been significant progress in the development of synthetic methods for inorganic NCs, enabling the precise tuning of their size, chemical composition, crystallinity, and shape, which is very important in controlling their properties. In addition to rendering unique material properties to individual NCs, the self-assembly of NCs provides a “bottom-up” approach for the fabrication of micro- or macroscale NC-based films with highly complicated nanostructures, which are difficult to achieve through conventional lithography-based fabrication processes. Moreover, NC building blocks enable solution-based, cheap, and scalable processes, which are highly beneficial for industrial applications.

One of the most interesting applications of NC building blocks is the colloidal self-assembly of NCs into ordered crystalline structures; that is, NC building blocks form various crystal structures, including face-centered cubic (fcc), body-centered cubic (bcc), and hexagonal close packed (hcp) structures, similar to how atoms or ions form crystalline structures [3,4]. More importantly, the use of two different types of NCs can yield highly ordered binary NC superlattices (BNSLs) with various packing structures, such as NaCl, MgZn_2_, and NaZn_13_, depending on the size and concentration ratio [5]. BNSLs exhibit not only structural diversity for tuning NC–NC interactions through the choice of NC constituents or packing symmetry, but also collective properties that are distinct from the sum of the individual characteristics. It is highly important to understand the self-assembly behavior of NCs for the development of novel materials because of their superior controllability in material design. In this review, we will broadly describe the self-assembly of colloidal NCs, including the fabrication method, formation mechanism of self-assembly, structural diversity of BNSLs, and mesoscale structure of self-assembled NC superlattices.

## 2. Self-Assembly of NCs

### 2.1. Methods for the Self-Assembly of NCs

**Figure 1:**
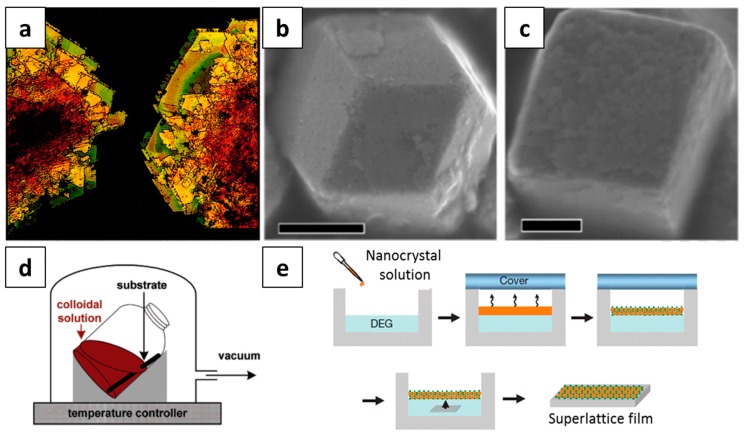
(**a**) Dark field optical micrograph of colloidal crystals formed by 2 nm CdSe nanocrystals (NCs) (Reproduced with permission from [10], Copyright American Association for the Advancement of Science, 1995). Scanning electron microscope (SEM) images of self-assembled supercrystals of (**b**) octahedral and (**c**) cubic Pt NCs (Reproduced with permission from [11]. Copyright American Chemical Society, 2013). Schematic illustration of the self-assembly of NCs by (**d**) the slow evaporation of NC solution under vacuum (Reproduced with permission from [12], Copyright American Chemical Society, 2006). and (**e**) liquid–air interface assembly (Reproduced with permission from [13]. Copyright Springer Nature, 2010). Scale bars in Figure 1b,c represent 500 nm and 200 nm, respectively.

Highly ordered NC superlattices can be prepared by the self-assembly of colloidal NCs. Mostly, colloidal inorganic NCs are synthesized by the high-temperature solvothermal decomposition process, which yields highly monodispersed NCs coated with alkyl chain ligands [6,7,8]. A few approaches have been adopted to build highly ordered NC superlattices with the prepared NC building blocks. One of the methods is simply drop-casting colloidal NCs in non-polar solvents such as hexane, toluene, or chloroform onto a solid substrate and allowing them to dry for a couple of minutes [9]. When the solvent evaporates, the NCs are densely solidified through various kinds of interactions including NC–NC interactions (i.e., van der Waals force and electrostatic interaction) and ligand–ligand interactions (i.e., hydrogen bonding). In addition to the simple drop-casting method, the recrystallization method has been used for the preparation of three-dimensional (3D) ordered NC superlattices by using a polar solvent to destabilize NC dispersion in a non-polar solvent, as shown in Figure 1a–c [10,11]. As the non-polar solvent slowly evaporates from the QD dispersion in the presence of the polar solvent, QDs start crystallizing because of their decreased solubility in the solution. Consequently, 3D micro-sized NC superlattices are formed because of van der Waals interactions between the NCs and the change in free energy during the crystallization process.

Another method for the self-assembly of NCs involves the slow evaporation of the solvent from the NC solution, inducing the crystallization of the NCs [12]. As shown in Figure 1d, a substrate (e.g., transmission electron microscope (TEM) grid or silicon wafer) is placed in a container with the NC solution. Then, the container is placed in a chamber and tilted by 60–70°. Next, the solvent is slowly evaporated under a low-pressure vacuum at 45 °C. As the concentration of NCs in the solution increases, it reaches the solubility limit of NCs in the solution, leading to the crystallization of NCs. Consequently, a well-ordered NC superlattice is formed on the substrate. A similar approach has been adopted to obtain NC superlattices at the liquid–air interface [13,14]. When NCs in non-polar solvents such as hexane and toluene are drop-casted on top of an immiscible polar solvent (e.g., ethylene glycol and diethylene glycol) in a well, followed by covering the top with a slide glass, the non-polar solvent on top of the liquid substrate slowly evaporates, and as the concentration increases, NCs are crystallized. Finally, a thin, long-range ordered NC film forms on top of the polar solvent, which is then transferred to a solid substrate for characterization. An advantage of the liquid–air interface self-assembly technique is that it yields uniform NC superlattice thin-films over a large area within a short time.

### 2.2. Self-Assembly of Spherical NCs

When self-assembled, NCs form highly ordered superlattices, resembling the atomic crystal structure; that is, self-assembled NCs can exhibit various packing symmetries including fcc and hcp, which have the highest packing density (74%), and non-close packing symmetries such as bcc and simple cubic (sc) symmetries with packing densities of 68% and 52%, respectively. When hard spheres assemble, preferably, packing occurs with the highest free volume entropy, leading to close-packed symmetries (i.e., fcc and hcp). Nonetheless, non-close packing symmetries, such as bcc and sc, are also often observed for NC superlattices, which is hard to explain on the basis of the entropy-driven assembly mechanism. Experimentally, it has been found that the softness of NCs, λ, which is defined as the extended ligand length-to-core radius ratio, is a very important factor that must be considered in the determination of the packing symmetries of NC superlattices [15]. This can be attributed to the interplay between entropic and enthalpic effects in fcc and bcc symmetries [16,17,18]. When *λ* is small—or in other words, when the ligand length is relatively short compared with the core radius—NCs act as hard spheres and preferably adopt an fcc symmetry due to its larger enthalpic gain compared to that of bcc. On the other hand, when *λ* is relatively large, NCs adopt a bcc symmetry because the entropic effects from ligand packing become dominant.

**Figure 2:**
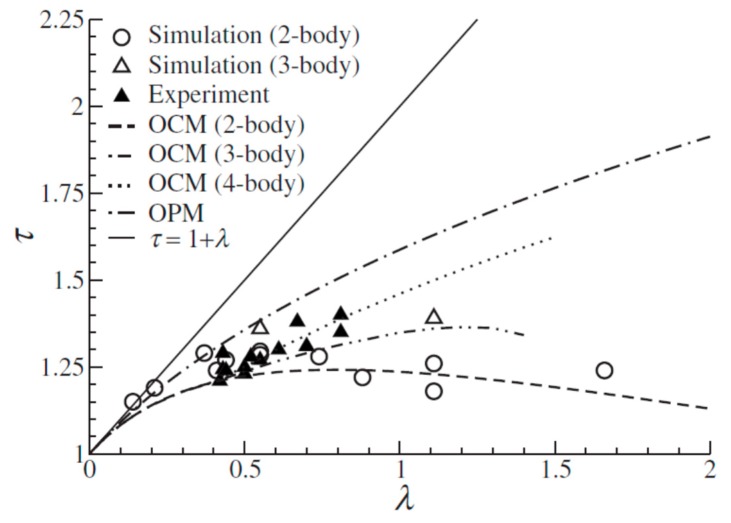
Scaled equilibrium distance *τ* vs. *λ*. When the surface ligands do not overlap each other, ***τ*** = 1 + ***λ***. Therefore, as the points or lines are more away from the solid line (*τ* = 1 + *λ*), it indicates more overlapped surface ligands. (Reproduced with permission from [20]. Copyright American Institute of Physics, 2009). OCM: Overlap cone model. OPM: Optimal packing model.

To interpret the softness-dependent self-assembly behavior of NCs, various kinds of theoretical models have been proposed. For example, on the basis of space filling between inorganic NC cores, two different models have been proposed: the optimal packing model (OPM) [19] and overlap cone model (OCM) [20]. The OPM postulates the densest packing of organic ligands along the NC core-to-core axis. The self-assembly behavior of NCs can be successfully predicted with the OPM using *λ* as a variable. For example, according to the OPM, the effective radius (*r*_i_) of NCs, which is half of the core-to-core distance, can be expressed as $r_{i}=R{\left(1+3\varepsilon \lambda \right)}^{\frac{1}{3}}$, where *R* is the inorganic NC core radius, *ε* is the ratio of the maximum surface area occupied by a ligand to the actual surface area covered, and *λ* is the ratio between the extended chain length to the core radius [19,21]. This formula can successfully describe not only the NC–NC separation distance but also the *λ* required for the transition from fcc to bcc. While the OPM fits three-dimensionally assembled NC superlattices well, the NC–NC separation determined using the OPM formula cannot be well applied to low-coordinated NC superlattices. To correct this flaw, the OCM has been proposed, wherein the truncated ligand cones intersect with each other and maximize the packing density, resulting in a shorter interparticle distance than that in the OPM. As shown in Figure 2, the OCM can successfully predict the interparticle distance between NCs in low coordination.

Later, the difference between the OPM and OCM was explained by Boles and Talapin, and they attributed it to the many-body effect in NC superlattices [22]; that is, because soft organic ligands can be deformed when there is another NC nearby, the NC–NC distance becomes smaller when NCs are surrounded by a low number of NCs. On the other hand, NCs in high coordination exhibit a longer NC–NC separation distance because of the limited deformability of the ligands, which shows good agreement with the OPM rather than with the OCM. The presence of the many-body effect in NC superlattices has led to the development of the orbifold topological model (OTM), which treats the deformable ligand coronas as topological defects [23]. The OTM predicts the formation behavior of BNSLs, the NC separation distance, and the stability of packing symmetries well.

**Figure 3:**
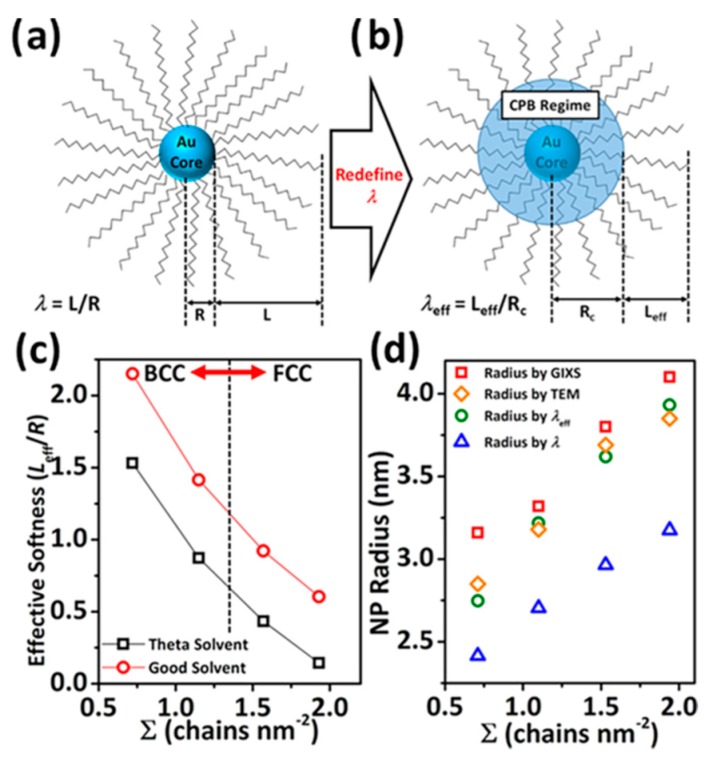
Illustrations of (**a**) the conventional concept of softness (*λ*) of nanoparticles (NPs) and (**b**) the concept of effective softness (***λ***_eff_). (**c**) Effective softness variation as a function of grafting density (Σ). (**d**) Comparison between the effective NP radius predicted by *λ* (blue triangle), *λ*_eff_ (green circle), and experimental results obtained from transmission electron microscopy (TEM, orange diamond) and grazing incidence x-ray scattering (GIXS, red square) (Reproduced with permission from [26]. Copyright American Chemical Society, 2019).

Alkyl-chain based ligands on NC surfaces have been replaced by other organic materials to diversify the phase diagram of NC superlattices. For example, polymeric ligands have been reported to offer enriched NC packing symmetries [24] as well as enhanced mechanical stability [25]. Moreover, polymeric ligand-coated NCs show a different self-assembly manner from that of conventional alkyl chain-coated NCs. For example, it is well-known that alkyl-chain-coated NCs preferentially adopt a bcc symmetry when *λ* is over 0.6–0.7 [15,16,19]. On the other hand, Yun et al. recently reported that Au@PS nanoparticles adopt fcc packing symmetries even at a *λ* of 3.0, which was attributed to the grafting density effect, wherein ligand penetration is limited around the NC surface, lowering the “effective softness” of the nanoparticles and thereby leading to the formation of assemblies with fcc symmetries. [26] The authors formulated the “effective softness”, *λ*_eff_, as a function of grafting density by including the concentrated polymer brush (CPB) regime as a part of the “hard core”, which was then applied to the OPM and successfully predicted the effective nanoparticle (NP) radius more accurately than the prediction by *λ*, as shown in Figure 3. Self-assemblies of DNA-coated NCs have been widely demonstrated [27,28]. DNA-based ligands can be designed to control the ligand–ligand interaction, which enables the programmable self-assembly of NCs. Dendrimers can also provide a wide range of interparticle spacing by changing the dendritic generation grafted on the NC surface [29,30].

### 2.3. Self-Assembly of BNSLs

**Figure 4:**
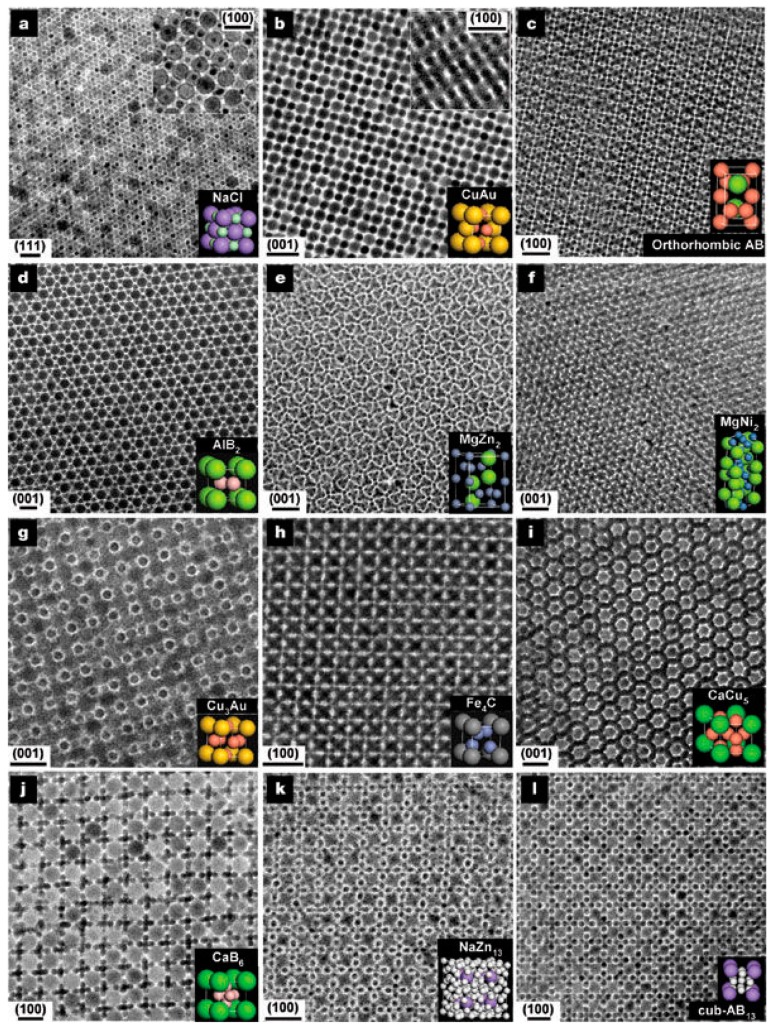
Structural diversity of binary nanocrystal superlattices (BNSLs). (**a**) 13.4 nm g-Fe2O3 and 5.0 nm Au NCs (NaCl-type), (**b**) 7.6 nm PbSe and 5.0 nm Au NCs (CuAu-type), (**c**) 6.2 nm PbSe and 3.0 nm Pd (AB-type), (**d**) 6.7 nm PbS and 3.0 nm Pd (AlB_2_-type), (**e**) 6.2 nm PbSe and 3.0 nm Pd (MgZn_2_-type), (**f**) 5.8 nm PbSe and 3.0 nm Pd (MgNi_2_-type), (**g**) 7.2 nm PbSe and 4.2 nm Ag (Cu_3_Au-type), h) 6.2 nm PbSe and 3.0 nm Pd (Fe_4_C-type), i) 7.2 nm PbSe and 5.0 nm Au (CaCu_5_-type), (**j**) 5.8 nm PbSe and 3.0 nm Pd (CaB_6_-type), (**k**) 7.2 nm PbSe and 4.2 nm Ag (NaZn_13_-type), and (**l**) 6.2 nm PbSe and 3.0 nm Pd (cub-AB_13_-type). Scale bars represent 20 nm (**a**–**c**,**e**,**f**,**i**–**l**) and 10 nm (**d**,**g**,**h**). (Reproduced with permission from [5]. Copyright Springer Nature, 2006).

In addition to single-component NC superlattices, highly ordered NC superlattices, called BNSLs, can be formed by using either a single type of NCs or a mixture of two different types of NCs with different sizes [31], shapes [5], and properties [32]. For example, Redl et al. demonstrated the formation of BNSLs using γ-Fe_2_O_3_ (magnetic) and PbSe (semiconducting) NCs with a precisely controlled size and narrow size distribution [33]. By varying the size ratio and concentration ratio of the NCs, they prepared BNSLs with various packing symmetries including AB_2_, AB_5_, and AB_13_. When the size ratio (*d*_PbSe_/*d***_γ_**_-Fe2O3_) was 0.58, AB_2_ and AB_13_ BNSLs were formed, and at a higher value of 0.63, AB_5_ BNSL was formed. This was a particularly interesting BNSL system because two different NCs with independently tunable optical (PbSe) and magnetic (γ-Fe_2_O_3_) characteristics were employed, which could enable the fine tuning of material properties. More importantly, BNSLs have enormous structural diversity, as demonstrated by Shevchenko et al., wherein 15 different BNSL symmetries, such as NaCl-, CuAu-, MgZn_2_-, MgNi_2_-, AlB_2_-, Cu_3_Au-, CaCu_5_-, and NaZn_13_-type (Figure 4), were observed [5]. BNSLs with various symmetries could be prepared using several types of NCs including Au, PbSe, Pd, Ag, and γ-Fe_2_O_3_ with different sizes.

The structure of BNSL thin-films are relatively stable in ambient conditions. There are studies which report that the thermal stability of NCs can be significantly enhanced upon the formation of BNSL compared with that of single-component NC films. For example, although FePt NCs are thermally unstable and easily sintered [34], it was reported that the BNSL structure consisting of FePt and MnO was preserved even after thermal annealing at 650 °C [35]. This could be attributed to the fact that the presence of thermally stable MnO NCs around FePt NCs spatially confine them to prevent coalescence. In addition to the thermal stability, the mechanical stability of BNSLs has been demonstrated, showing that the BNSLs can form free-standing membranes [13] and even monolayers [36]. Also, the membranes are robust enough to enable pattern transfer [37].

**Figure 5:**
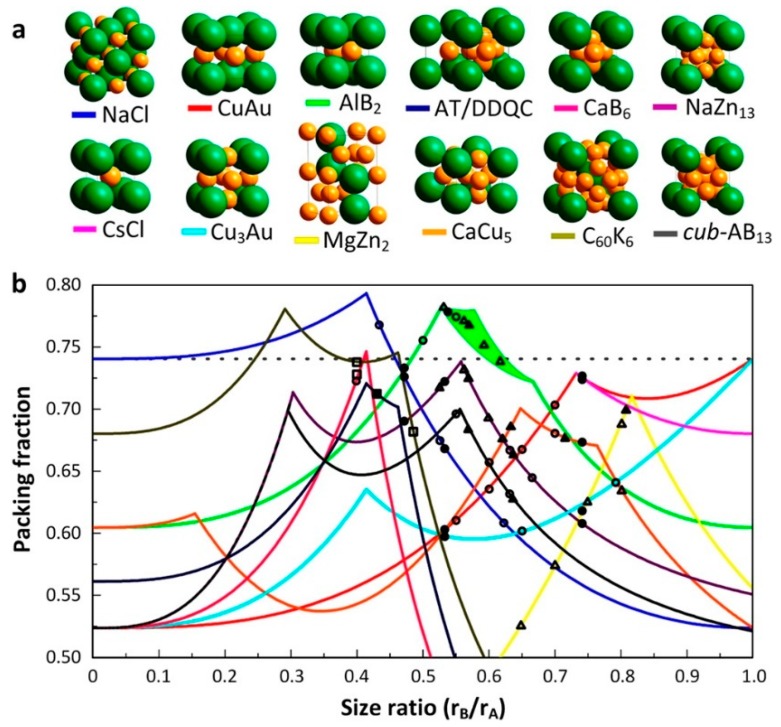
(**a**) Illustrations of different types of BNSL packing symmetries. (**b**) Phase diagram showing the packing fraction of BNSLs as a function of size ratio. (Reproduced with permission from [22]. Copyright American Chemical Society, 2015).

To understand the formation mechanism of BNSLs, the change in the free energy of the system must be taken into account. The free energy change is determined by NC–NC interactions as well as the entropic change during the formation of BNSLs. In addition, as reported, during the formation of opal crystals by micron-sized colloidal particles, the NCs self-assemble into well-ordered superlattices even without the presence of NC–NC interactions, which is called “entropy-driven self-assembly”. When colloidal particles form well-ordered arrays with the highest packing fraction, the system obtains an additional free volume, which eventually leads to maximum entropy. In addition to the entropy-based principle, the “space-filling principle”, which was proposed by Murray and Sanders [38], can be used to describe the self-assembly behavior of BNSLs. For single-component hard spheres, the highest packing symmetry is either fcc or hcp (both have a filling fraction of 0.74). When NCs with two different sizes are assembled, BNSLs are formed with a packing fraction of over 0.74, which is thermodynamically more stable than close-packed symmetries (i.e., fcc and hcp). Therefore, according to the space-filling principle, targeted BNSL structures can be obtained by tuning the size ratio between two different NCs. Figure 5 presents the phase diagram of BNSLs based on the space-filling principle. As observed, each BNSL symmetry shows different packing fractions. Moreover, it has been experimentally demonstrated that two different NCs can assemble into BNSLs with packing fractions higher than those of close-packed structures (0.74), as shown in the phase diagram.

It has been reported that BNSLs with both translational and rotational orders, and with rotational order but without translational order, can be formed, which are called quasicrystals [39]. For example, when a combination of 5 nm Au NCs and 13.4 nm Fe_2_O_3_ NCs was used for self-assembly, the NCs formed BNSLs with a 12-fold rotational symmetry but without any translational symmetry (dodecagonal quasicrystals). In this case, the size ratio was 0.34, wherein the CaB_6_ and AlB_2_ BNSLs have the same packing fraction, indicating that both structures are thermodynamically stable. AlB_2_ and CaB_6_ BNSLs consist of triangular and square tiles, respectively, whereas quasicrystal structures contain periodic arrays of triangular and square tiles. Therefore, when the size ratio of 0.34 was used, dodecagonal quasicrystals were formed through the periodic arrangement of BNSLs with both symmetries (AlB_2_ and CaB_6_).

**Figure 6:**
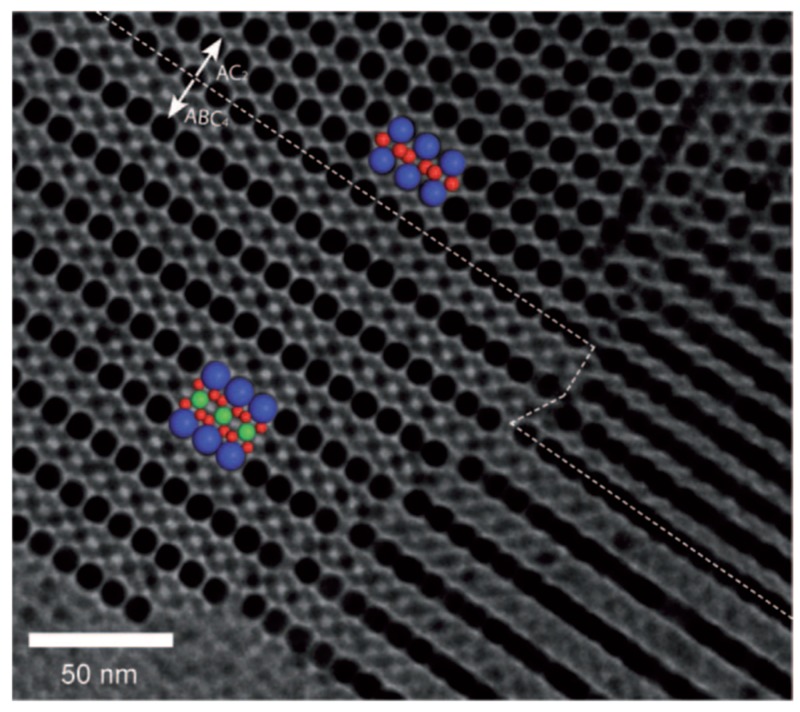
Transmission electron microscopy (TEM) image of the AlMgB_4_-type ternary superlattice (PbSe(l)-PbSe(m)-CdSe(s)_4_ nanocrystal superlattice, l = large, m = medium, and s = small) in epitaxial contact with the AlB_2_-type binary superlattice (PbSe(l)-CdSe(s)_2_). The TEM image and schematic show the (100) planes of the ternary superlattice, in which PbSe (l, blue spheres), PbSe (m, green spheres), and CdSe (s, red spheres) can be individually observed. (Reproduced with permission from [40]. Copyright John Wiley and Sons, 2009.

In addition to BNSLs, more complicated NC superlattices can be formed using more than two types of NCs. For instance, in 2009, Vanmaekelbergh et al. demonstrated the formation of well-ordered ternary NC superlattices using 12.1 nm PbSe NCs (A), 7.9 nm PbSe NCs (B), and 5.8 CdSe NCs (C): ABC_4_ (isostructural with AlMgB_4_) along with AB_2_ (AlB_2_) and BC_2_ (MgZn_2_) [40]. The detailed structure of the ternary NC superlattice was confirmed by 3D electron tomography, as shown in Figure 6. Interestingly, the packing fraction of the AlMgB_4_ ternary NC superlattice was 0.64, which was lower than those of AlB_2_ (0.76) and MgZn_2_ (0.67). This was attributed to the fact that the ternary NC superlattice was obtained not by the effects of entropy but rather by the combination of NC–NC interactions and thermodynamic factors. To predict the most stable packing symmetry of NC superlattices, the total energy must be calculated.

**Figure 7:**
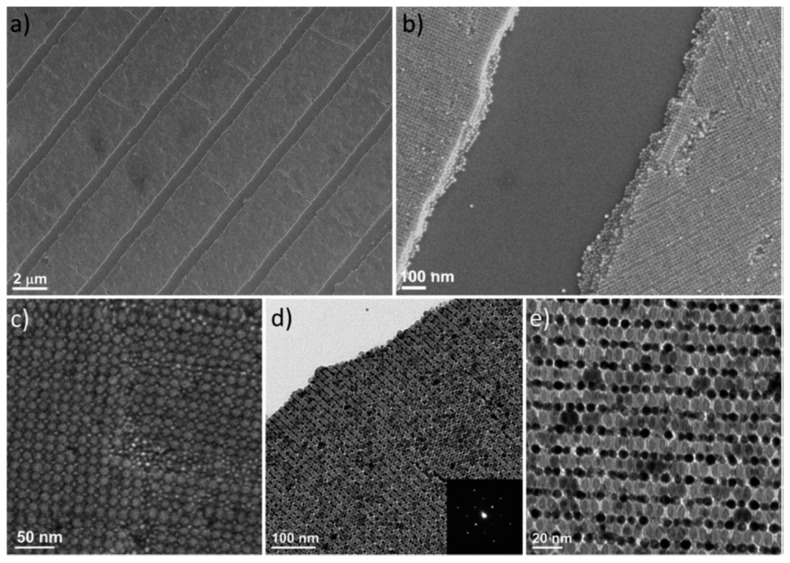
Hierarchical BNSL nanostructures formed by liquid–air interfacial assembly and transfer printing. (**a**–**c**) SEM images, and (**d**) low-magnification (inset: selected-area electron diffraction pattern) and (**e**) high-magnification TEM images of patterned AlB_2_ BNSLs assembled from FeOx and Au NCs (Reproduced with permission from [37]. Copyright American Chemical Society, 2017).

Generally, BNSLs are grown as 2D thin films, depending on the fabrication method. In 2015, Murray and Kagan et al. reported the fabrication of multiscale-patterned BNSLs by colloidal self-assembly and transfer printing [37]. BNSL thin films are first fabricated from NC building blocks of various materials such as metals, semiconductors, magnetics, and dielectrics by the liquid–air interfacial assembly method; the BNSL thin films formed at the interface are then transferred onto patterned polydimethylsiloxane (PDMS) molds by the Langmuir–Schaefer technique. During this process, only the BNSL thin film on the raised region of the PDMS pattern is transferred; thus, patterned BNSL nanostructures are obtained on the substrate. The transferred structures exhibit a mesoscale order, while the BNSLs maintain the nanoscale order, resulting in multiscale hierarchical architectures. Figure 7 shows the TEM and SEM images of a patterned BNSL film obtained by self-assembly and transfer printing. The SEM images reveal the formation of mesoscale line patterns. The TEM images reveal that the AlB_2_-type BNSL of Au and FeOx NCs is maintained after transfer printing, exhibiting long-range order over a large area. The nanoscale BNSL structures can be readily tuned by changing the size and composition of the NC building blocks, as previously described. In addition, the mesoscale pattern (circular or square arrays) of BNSLs can be tailored by changing the shape of the PDMS molds. Moreover, the BNSL patterns can be stacked by layer-by-layer transfer printing, indicating that the fabrication of complex BNSL structures is possible by sequential self-assembly and multiple transfer printing.

**Figure 8:**
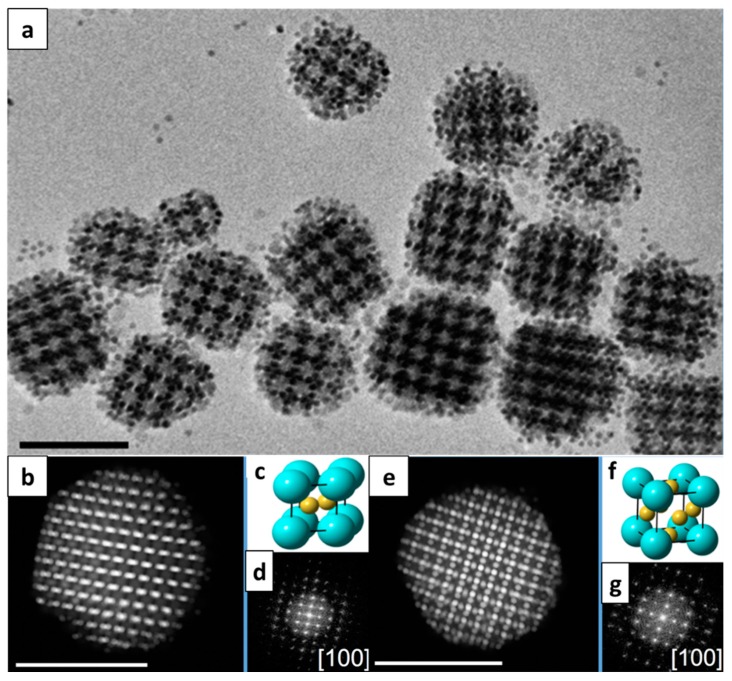
(**a**) A TEM image of ico-NaZn_13_-type supracrystals. High-angle annular dark field scanning TEM (HAADF-STEM) images of (**b**) AlB_2_-type supracrystals and (**e**) AuCu_3_-type supracrystals. (**c**,**f**) The unit cells of the AlB_2_-type lattice and AuCu_3_-type lattice, and (**d**,**g**) their fat Fourier transformation (FET) patterns. The scale bar in all the TEM images is 100 nm. (Reproduced with permission from [41]. Copyright American Chemical Society, 2018)

BNSL can be formed not only into 2D thin films but also into a 3D confined emulsion. For example, Wang et al. reported the formation of colloidal BNSL supracrystals with various symmetries, as shown in Figure 8 [41]. They demonstrated the preparation of BNSL supracrystals by oil-in-water emulsion droplets of Au and Fe_3_O_4_ NCs followed by the evaporation of the oil phase, which induces the co-crystallization of NCs into 3D confined BNSL structure. By controlling the ratio between Au and Fe_3_O_4_ NCs, various BNSL symmetries such as AB_2_, AB_3_, and AB_13_-type symmetries could be achieved. Recently, such 3D confined BNSL supracrystals of CoFe_2_O_4_-Fe_3_O_4_ have been reported to have superior lithium storage properties compared with their single-component counterparts [42]. The enhanced electrochemical properties were attributed to the non-close packed symmetry of the BNSL supracrystals, which promoted better mass transport as well as endurance against volumetric changes during lithiation and delithiation processes.

### 2.4. Self-Assembly of Anisotropic BNSLs

Various types of anisotropic NCs including rods, cubes, tetrahedrons, and plates have been reported. Anisotropic NCs have received particular attention in optics [43], magnetics [44], catalysis [45], and electronics. [46] Accordingly, considerable efforts have been made to prepare NC superlattices using anisotropic NCs as building blocks, thereby maximizing the potential application of NCs. CdSe nanorods are one of the most extensively studied anisotropic NCs for self-assembly because of their shape-dependent light polarization properties. According to Talapin et al., when 1D CdSe nanorods are self-assembled by controlling the dispersibility of nanorods, either nematic or smectic liquid crystals are formed. [47] The formation of a long-range nanorod superstructure was attributed to a combination of strong side-to-side van der Waals interactions, antiparallel side-by-side dipole pairing, and entropy effects, which led to an increase in the free volume space, thereby achieving the highest packing density. The self-assembly of anisotropic NCs of various materials including Au, Cu_x_S, LaF_3_, β-NaYF_4_, and GdF_3_ has been reported [48,49,50,51,52].

In addition to the superlattices of single components, the formation of BNSLs of multicomponent anisotropic NCs has also been reported. For instance, in 2006, the formation of BNSLs was demonstrated using LaF_3_ triangular nanoplates (9 nm side) and 5 nm spherical Au NCs [5]. Also, AB_2_-type BNSLs could be also fabricated by the combination of Fe_3_O_4_ NCs and spherical β-NaYF_4_ nanorods as building blocks [53]. The experimentally obtained results were compared with Monte Carlo simulation results, and it was found that the ligand–ligand interaction and depletion attraction caused by the extra ligands around the NCs affected the formation of BNSLs of anisotropic NCs. In addition, on the basis of the space-filling principle and size ratio-dependent self-assembly behavior, it was found that entropy-driven free energy maximization determined the BNSL symmetry, leading to the formation of BNSL structures with the highest packing density.

**Figure 9:**
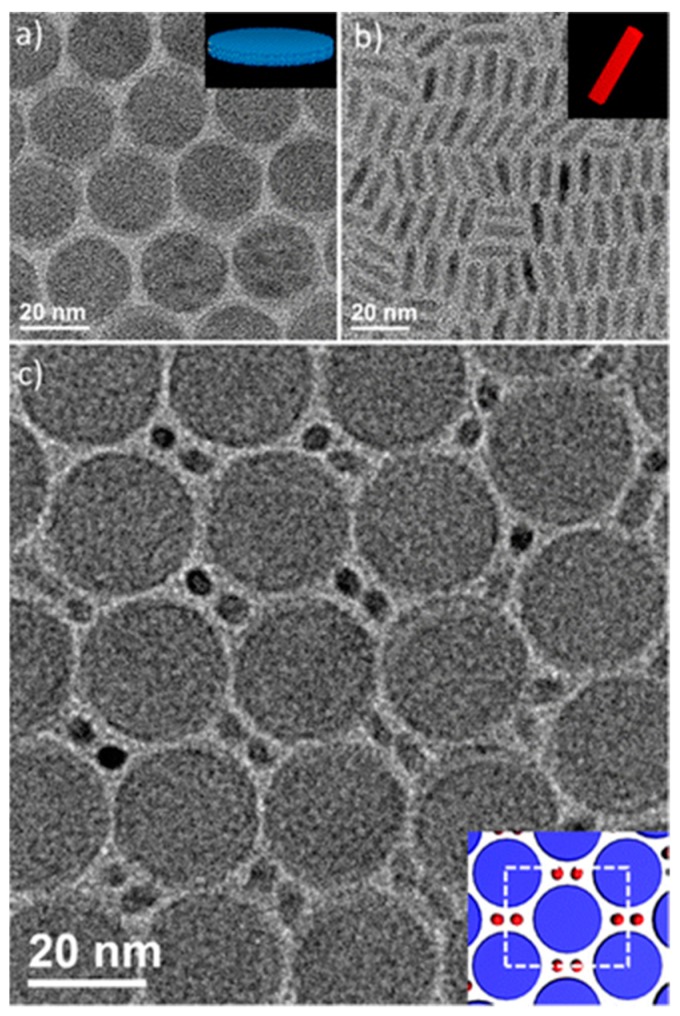
TEM images of binary superlattices of two anisotropic NC building blocks. (**a**) LaF_3_ nanodisks, (**b**) CdSe/CdS nanorods, and (**c**) AB_2_ BNSLs of LaF_3_ nanodisks and CdSe/CdS nanorods. Inset is the illustration of the BNSL structure. Reproduced with permission from [54]. Copyright American Chemical Society, 2015.

Moreover, BNSLs consisting of two different anisotropic NCs, LaF_3_ nanodisks (2 nm thickness and 15–25 nm diameter) and CdSe-CdS nanorods, have been reported [54]. In this work, NCs dispersed in hexane were drop-casted on top of diethylene glycol (immiscible with hexane) and slowly dried at the liquid–air interface, yielding highly ordered BNSL structures. In particular, because of the shape anisotropy of these NCs, they showed shape-selective interactions. The nanodisks self-assembled to form a stacked columnar structure through face-to-face van der Waals interactions, yielding a 2D hexagonally packed liquid crystalline structure, as presented in Figure 9a. On the other hand, the 1D nanorods assembled to form smectic lamellar liquid crystalline structures via side-by-side van der Waals interactions (Figure 9b). Moreover, during the self-assembly of the two different anisotropic NCs, the nanorods vertically aligned to fill the interstitial sites between the hexagonally packed columnar structure of the nanodisks, filling the hierarchically assembled BNSL structure, as shown in Figure 9c. It was found that AB-, AB_2_-, and AB_6_-type BNSL structures could be formed depending on the size and concentration ratio between the nanodisks and nanorods. This phenomenon further confirms that the BNSL structure is formed to maximize the packing fraction of NCs in the system, similar to the formation principle of spherical BNSL structures. This reveals a particularly interesting aspect of the formation of anisotropic NC-based BNSLs: the entropic factor affects the final packing symmetry of BNSLs of anisotropic NCs even while the orientation is controlled. This result indicates that the formation of BNSLs of anisotropic NCs is more complicated; therefore, the directional order, van der Waals interactions between the NCs, and entropic effects must be taken into consideration. In addition to BNSLs, ternary NC superlattices comprising three different anisotropic NCs (i.e., nanorods and two different nanodisks) were reported by the authors.

Interestingly, anisotropic NCs can be self-assembled through selective interaction, depending on their shape, in a similar way to puzzles, which was previously demonstrated in lock and key colloids. In 2013, a shape-complementary BNSL structure comprising GdF_3_ rhombic nanoplates and Gd_2_O_3_ tripodal nanoplates was reported [55]. In this case, the side length and interior angle of the rhombic nanoplates were precisely controlled to be close to those of the tripodal nanoplates. Subsequently, the two different anisotropic NCs were assembled into interlocked BNSLs on the basis of shape complementarity. This work demonstrates that the self-assembly of shape-complementary anisotropic building blocks may provide a unique design rule to direct the formation of BNSL thin films over a large area with high complexity in a predictable way.

## 3. Perspectives

The self-assembly of colloidal NCs offers significant potential for sophisticated, novel material design because of their structural diversity and the variety of material choices, as well as the formation of complicated structures. Since NC–NC interactions can be more effectively tuned through the formation of various types of BNSL structures than those of single-component NC superlattices, a larger variety of novel material characteristics can be achieved. Many reports have been published describing the methods of formation, the mechanism, and the structural characterization of NC superlattices. Although structure dependent, synergistic collective interactions between NCs in BNSLs are reported in electronics [56,57,58], optics [59], catalysis [32], and magnetics [56]; however, a limited number of studies on the intrinsic material properties of highly ordered NC superlattices, particularly the BNSLs, have been performed so far.

To achieve the full potential of BNSL-based materials, it is necessary to obtain an in-depth understanding of complicated NC–NC interactions within various BNSL structures. For example, some NC–NC interactions can occur over a few nanometers, while energy or electron transfer between NCs exponentially decreases due to the presence of surface ligands on NC surfaces. Therefore, it is important to reduce the distance between NCs while preserving the BNSL structures in order to enhance the interaction between NCs. The development of a fabrication method of BNSLs using NCs which their ligands are stripped or exchanged to short-chain ligands will enable BNSLs to be applied in many emerging fields. Moreover, through the multiscale fabrication method of BNSLs, synergistic effects between mesoscale structural effects and collective properties of BNSLs may be realized. For examples, mesoscale patterns of BNSLs may add mesoscale photonic effects to the collective properties of BNSLs. In addition, BNSL supracrystals may be utilized in the application of biomedical imaging agents.

In terms of processing, it is imperative to develop large-area-BNSL formation techniques for the commercialization of BNSL-based novel materials. Although the facile and high-throughput fabrication method of large-area BNSL structures are demonstrated [60], there are many experimental difficulties to forming large-area, uniform superlattice thin-films. Therefore, the undertaking of systematic studies to understand the effect of self-assembly conditions, including the choices of the building blocks and the concentration of NCs on the formation of defect-free, long-range ordered BNSLs, would be important. If the fabrication of uniform large-area NC thin films can be realized, more accurate material analysis can be performed to identify BNSLs with outstanding material properties, eventually promoting the application of NC thin films for the development of novel materials in several industrial fields.
